# Treatment of Hiccough

**Published:** 1848-01

**Authors:** 


*Treatment of Hiccough.*—Dr. Boyer relates three cases of prolonged and alarming hiccough, which, having resisted all the usual means employed for its relief, were relieved by the application of pressure, a practice first suggested by Bordeu and since revived by M. Rostan. A large pad is laid on the epigastrium, and bound forcibly on by means of a towel or bandage. It generally causes instant relief, but if discontinued too soon the hiccough returns. It is usually necessary to wear it for twenty-four hours, before it can be safely removed.—*Revue Medico-Chirurgicale*, July.

